# Development of a modified head and neck quality assurance phantom for use in stereotactic radiosurgery trials

**DOI:** 10.1120/jacmp.v14i4.4313

**Published:** 2013-07-08

**Authors:** Austin M. Faught, Stephen F. Kry, Dershan Luo, Andrea Molineu, David Bellezza, Russell L. Gerber, Scott E. Davidson, Walter Bosch, Robert E. Drzymala, Jim Galvin, Robert Timmerman, Jason Sheehan, Michael T. Gillin, Geoffrey S. Ibbott, David S. Followill

**Affiliations:** ^1^ Department of Radiation Physics The University of Texas MD Anderson Cancer Center Houston TX; ^2^ The University of Texas Graduate School of Biomedical Sciences at Houston Houston TX; ^3^ St. Luke's Radiation Therapy and CyberKnife St. Luke's Episcopal Hospital Houston TX; ^4^ Department of Radiation Oncology Saint Francis Hospital Tulsa OK; ^5^ Deparment of Radiation Physics The Methodist Hospital System Houston TX; ^6^ Department of Radiation Oncology Physics Division Washington University School of Medicine Saint Louis MO; ^7^ Department of Radiation Oncology Thomas Jefferson University Hospital 111 South 11th Street Philadelphia PA; ^8^ Department of Radiation Oncology The University of Texas Southwestern Medical Center Dallas TX; ^9^ Department of Neurological Surgery University of Virginia Charlottesville VA USA

**Keywords:** quality assurance, head and neck phantom, stereotactic radiosurgery, TLD, film dosimetry, I. INTRODUCTION

## Abstract

An anthropomorphic head phantom, constructed from a water‐equivalent plastic shell with only a spherical target, was modified to include a nonspherical target (pituitary) and an adjacent organ at risk (OAR) (optic chiasm), within 2 mm, simulating the anatomy encountered when treating acromegaly. The target and OAR spatial proximity provided a more realistic treatment planning and dose delivery exercise. A separate dosimetry insert contained two TLD for absolute dosimetry and radiochromic film, in the sagittal and coronal planes, for relative dosimetry. The prescription was 25 Gy to 90% of the GTV, with ≤10% of the OAR volume receiving ≥8Gy for the phantom trial. The modified phantom was used to test the rigor of the treatment planning process and phantom reproducibility using a Gamma Knife, CyberKnife, and linear accelerator (linac)‐based radiosurgery system. Delivery reproducibility was tested by repeating each irradiation three times. TLD results from three irradiations on a CyberKnife and Gamma Knife agreed with the calculated target dose to within ± 4% with a maximum coefficient of variation of ±2.1%. Gamma analysis in the coronal and sagittal film planes showed an average passing rate of 99.4% and 99.5% using ±5%/3mm criteria, respectively. Results from the linac irradiation were within ±6.2% for TLD with a coefficient of variation of ±0.1%. Distance to agreement was calculated to be 1.2 mm and 1.3 mm along the inferior and superior edges of the target in the sagittal film plane, and 1.2 mm for both superior and inferior edges in the coronal film plane. A modified, anatomically realistic SRS phantom was developed that provided a realistic clinical planning and delivery challenge that can be used to credential institutions wanting to participate in NCI‐funded clinical trials.

PACS number: 87.55 ‐v

I.

It has been shown that pituitary adenomas, such as acromegaly, can be safely and effectively treated with stereotactic radiosurgery (SRS).^1^, ^2^ Frequently used radiosurgery systems for treating such brain lesions include Gamma Knife,^1^, ^3^, ^4^, ^5^ CyberKnife,^1^, ^6^, ^7^ and linear accelerator (linac)‐based treatments.^1^, ^8^, ^9^ Because each system involves higher‐than‐normal doses per fraction and steep dose gradients,^2^, ^8^ there is an increased importance to accurately delivering the treatment to the patient.^(2)^ Otherwise, the consequences can be devastating, as recently reported in the press.^10^, ^11^, ^12^, ^13^, ^14^, ^15^, ^16^, ^17^ It has been found that the most effective means of reducing the likelihood of a medical event (defined as a deviation in delivered dose by greater than 10% from the prescription dose) is to attempt to reduce human errors involved in the radiation treatment.^(18)^

In assessing the quality of the overall treatment procedure, patient‐image acquisition, treatment planning, and dose delivery should all be considered. As a result, a simple homogenous phantom may not be adequate.^(19)^ By designing a humanoid‐shaped phantom with critical structures and heterogeneities, the overall treatment procedure may be more effectively evaluated.^19^, ^20^

Currently, for quality assurance and credentialing institutions in clinical trials involving SRS, the Radiological Physics Center (RPC) uses an anthropomorphic head phantom with a simple, spherical target of diameter 1.9 cm. Treatment criteria and SRS guidelines were established by the RPC in conjunction with the Radiation Therapy Oncology Group (RTOG) to assess an institution's ability to adequately deliver an SRS treatment.^(21)^ Phantom results are evaluated based on the following four criteria: 1) ratio of measured dose to reported dose at the center of the target to be 1.00±0.05; 2) ratio of measured treated volume to reported treated volume falls within the range of 0.75–1.25; 3) minimum dose to the target is ≥90% of the prescription dose; and 4) treated volume to target volume ratio is between 1.00 and 2.00.^(21)^ During phantom evaluation, the reported treated volume is determined from the prescription isodose line reported by the institution's treatment planning system. The measured treated volume is an ellipsoid, with diameters determined from the location of the prescription dose located on the orthogonal film profiles. The target volume is that corresponding to the 1.9 cm diameter spherical structure in the imaging insert of the phantom.

It has been reported that even with a single simple target, the majority of institutions irradiating this phantom fail to meet all four criteria,^(21)^ indicating a need for this QA tool to improve patient treatments.^(21)^

With input from the RTOG and Advanced Technology Consortium (ATC), it was determined that there was a need for a more rigorous test in credentialing institutions participating in SRS clinical trials. To address that need, the phantom commissioned in this study was a redesigned form presented by Balter et al.^(22)^ As detailed by Molineu et al.,^(20)^ these anthropomorphic phantoms need to be lightweight and durable so that they may be inexpensively mailed to institutions; the outer shell should be human‐like so that it mimics realistic anatomic clinical setups; they should contain a target volume and organs at risk; and they should be capable of holding dosimeters to verify the accuracy of the plan delivery.^(20)^

The aim of this study is to address the need for a more rigorous test in credentialing institutions that wish to participate in SRS clinical trials. To that end, a phantom was designed, developed, and tested for reproducibility to assess the overall usefulness of the phantom as a credentialing tool for SRS delivery by a Gamma Knife radiosurgery system, CyberKnife robotic radiosurgery system, and standard linac‐based radiosurgery system.

## II. MATERIALS AND METHODS

### A. Phantom design

**Figure 1:**
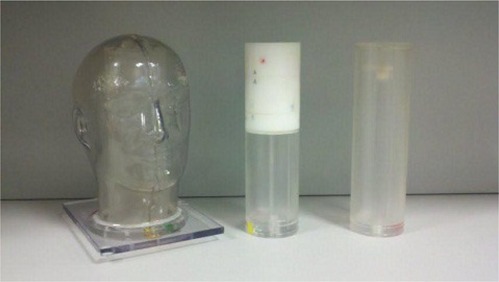
The anthropomorphic outer shell to the phantom (left), the dosimetry insert containing radiochromic film and TLD (middle), and the modified imaging insert containing the target and OAR (right) are shown.

The anthropomorphic outer shell, as shown in Fig. 1, was constructed of hollow plastic and supplied by The Phantom Laboratory (Salem, New York). The realistic size and human‐like features of this outer shell allow for the use of most stereotactic immobilization devices. At the base of the phantom, a PVC pipe and fitting are used for filling the hollow phantom with water. To minimize the accumulation of air bubbles within the filled shell, a small hole near the base allows for air to escape while filling the phantom shell. A cylindrical recess in the base extends into the shell until just below the phantom head's crown. This allows for the insertion of cylindrical imaging and dosimetry inserts, shown to the right of the head shell in Fig. 1.

**Figure 2:**
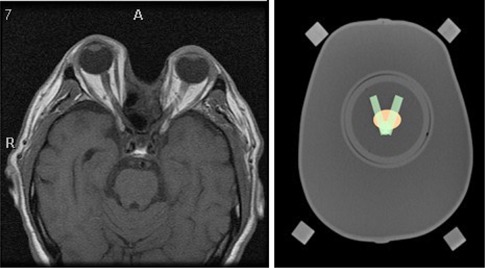
Transverse view of patient MRI (left) with optic apparatus and transverse view of phantom with proposed target (red) and OAR (green).

Modifications to the original SRS phantom design, which contained only a spherical target in the imaging insert, included changing the target shape to be an ellipsoid (based on the pituitary) measuring 2.5 cm, 1.9 cm, and 1.3 cm in the AP, left/right, and SI directions, respectively, and adding an adjacent (2 mm superior) organ at risk (OAR) (optic chiasm). The V‐shaped OAR measured 0.65 cm thick in the SI direction, 0.8 cm at the base of the V, and 2.5 cm at the widest part of the V in the left/right direction. The extent of the OAR along the AP direction was 3.85 cm. These organs simulated those encountered when treating acromegaly, as seen in Fig. 2. The target and OAR proximity provided a more realistic treatment planning and dose delivery exercise than the original single spherical target design. Similar to the outer shell, the imaging insert was constructed to be hollow and watertight to reduce weight, while still allowing for tissue‐like conditions during the imaging process. A separate dosimetry insert placed within the head recess during treatment delivery contained two thermoluminescent dosimeters (TLD) for point dosimetry comparison, and radiochromic film in the sagittal and coronal planes for 2D dosimetry, similar to that described previously by Balter et al.^(22)^ and Amador et al.^(21)^

### B. Phantom dosimeters

As the means for evaluating point‐based dosimetry, TLD‐100 powder, in small capsules, was placed into the dosimetry insert used during irradiation. With respect to the center of the target, the TLDs are 3 mm left, 3 mm posterior, and 2.5 mm superior and 3 mm right, 3 mm anterior, and 2.5 mm inferior, respectively. The capsules are about 15 mm in height and 5 mm in diameter, and have a 1 mm wall thickness. Each capsule contained about 20 mg of TLD powder, enough for a single reading.

After irradiation, TLDs were read by using an established reading procedure.^(23)^ Briefly, this involves a two‐stage calibration process that corrects for batch specific properties (e.g., dose‐response linearity, energy dependence, and fading corrections) and TLD reader system constancy. The latter calibration is done by irradiating TLD to a known dose from a cobalt‐60 system in order to determine the system sensitivity. Periodic checks during readout sessions verify reader consistency. The final TLD reading has a reported uncertainty of ±2%.^23^, ^24^

As a means of measuring 2D dose distributions, radiochromic films (GAFCHROMIC MD‐55 film; Nuclear Associates, Carle Place, NY) were used. The high spatial resolution, low spectral sensitivity, tissue equivalence, and lack of angular dependence all made the film a good dosimeter for steep dose gradients found in SRS plans. Two sheets of film were oriented in the sagittal and coronal planes of the phantom and were located such that the intersection of the film closely corresponded to the center of the target as viewed from the transverse cross section. Each film sheet measures approximately $6\times 6\;{\text{cm}}^{2}$. Six small holes in the dosimetry insert allowed for each film to be pricked with a needle in order to ensure proper spatial registration with respect to the phantom and treatment plan data.

After irradiation, all films were stored together such that they had the same temperature, light exposure, and background radiation levels. Reading of film was performed using a 633 nm laser CCD transmission densitometer (Personal Densitometer SI; Molecular Dynamics, Sunnyvale, CA) with a scanning resolution of 0.1 mm. A mask to reduce light contamination was placed around each film during scanning. A dose response curve for the batch of film used was determined to correct for nonlinearities in the film's dose response. All irradiated films were from the same lot of film. Each film was saved as a 12‐bit tagged image film format file, and an average background from an unexposed piece of film was subtracted before film analysis.

During analysis, the film's dose distribution was normalized to the measured TLD dose in the phantom.

### C. reproducibility studies

CT images obtained with the imaging insert in place were used to create a treatment plan that met the dose prescription and normal tissue constraints as specified by a proposed acromegaly clinical trial. However, the clinical trial constraints were modified for this phantom to make the objective achievable for the phantom anatomy. Specifically, the dose constraint to the optic chiasm was modified from ≤1% of the OAR volume receiving 8 Gy to being ≤10% of the OAR volume receiving 8 Gy. The phantom dose limits alongside the proposed clinical trial specifications are listed in Table 1.

**Figure 3:**
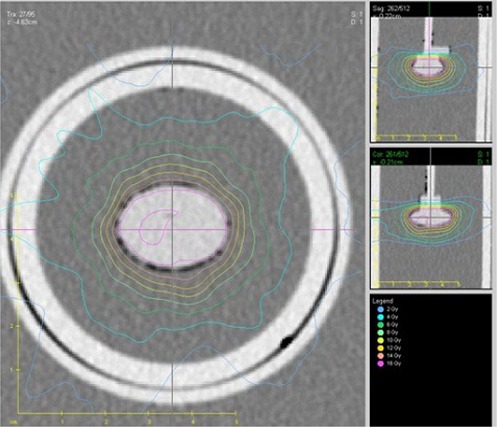
Isodose lines in the axial (left), sagittal (top right), and coronal (bottom right) planes at isocenter, taken from the CyberKnife treatment plan are shown as an example of a treatment plan that meets the established credentialing criteria.

Three different treatment plans, adhering to the agreed dose specifications listed in Table 1 were generated, one for each of three treatment delivery systems: a Gamma Knife radiosurgery system (Elekta AB, Stockholm, Sweden), CyberKnife robotic radiosurgery system (Accuray, Sunnyvale, CA), and linac delivery using a Varian Novalis machine (Varian Medical Systems, Palo Alto, CA). Plans for the three modalities were created using Leksell GammaPlan v8.3.1, MultiPlan v3.5.4 (Elekta), and BrainLAB Novalis Treatment Planning v5.32 (BrainLAB AG, Feldkirchen, Germany), respectively. The phantom was irradiated three consecutive times with minimal disturbance of phantom positioning when exchanging dosimeters between irradiations for each plan to test for reproducibility and dose delivery accuracy. Plans consisted of a 15‐shot Gamma Knife plan, an 87‐node 190 beam CyberKnife plan, shown in Fig. 3, and a two‐conformal arc linac‐based plan. CT slice thicknesses used in treatment planning were 1.00 mm for Gamma Knife and linac‐based plans, and 1.25 mm for the CyberKnife plan. Gamma Knife and linac deliveries used a Leksell stereotactic head frame and BrainLAB frame less immobilization device, respectively, while CyberKnife utilized orthogonal kV images for position verification before and throughout delivery. The TLDs from each irradiation were read two weeks after irradiation. Point comparisons of treatment plan calculated doses at the location of the TLD and measured dose data were done using the average of the two measured to reported dose ratios. A 2D gamma analysis comparing the calculated dose distribution to the measured dose distribution using the film dose distribution normalized to the TLD doses was performed. Gamma analysis was performed using an in‐house program executed in MATLAB (The MathWorks Natick, MA). When an institution's TPS does not allow for the plan to be exported in DICOM/ RT format, an equally acceptable means of 2D analysis is measuring distance to agreement from dose profiles. For this reason, in evaluating the linac‐based plan, the dose planes of interest were exported as ASCII files and compared to film manually in MATLAB. The reported DTA was the average from three measurements at 0.25, 0.50, and 0.75 of the maximum TPS‐calculated dose along the profile.

**Table 1:** Dose prescription and constraint specifications for treatment of pituitary adenoma

*Structure*	*Trial Specifications*	*Phantom Specifications*
Pituitary Adenoma	25 Gy to at least 90% of GTV	25 Gy to at least 90% of GTV
Optic Apparatus (chiasm and optic nerves)	<10Gy maximum dose (0.01 cc) ≤1% volume receiving 8 Gy	<10Gy maximum dose (0.01 cc) ≤10% volume receiving 8 Gy

### D. Dosimeter registration

In order to perform the gamma analysis and compare reported TPS calculated data with the measured doses, the positioning of the phantom film dosimeters was registered with the coordinate system of the TPS dose distribution. The film registration was performed using six pinpricks made in the radiochromic film prior to irradiation and related to the known coordinate of each pinprick relative to the center of the dosimetry insert. By registering the pinpricks during film analysis and comparing the distances between points to measured values, the phantom was successfully registered with a high degree of reproducibility. Multiple measurements demonstrated registration agreement for all irradiations to be within 0.4 mm.

### E. Verification of phantom geometry

Because the TPS‐calculated data are registered to the imaging insert and the film is registered to the dosimetry insert, it is necessary to ensure proper spatial relationship between the two different inserts. The phantom was imaged using an AcQsim CT scanner (Phillips Healthcare, Amsterdam, The Netherlands) with a Leksell stereotactic frame (Elekta) for immobilization. Using the generated images at a 1 mm slice spacing from scans with both the dosimetry insert and imaging insert in place, the positional relationship of the center of the target in the imaging insert and the intersection of the films within the dosimetry insert was determined.

## III. RESULTS & DISCUSSION

### A. Verification of phantom geometry

The agreement between the dosimeter and imaging inserts was found to be within 0.2 mm in the lateral direction, 0.2 mm in the anteroposterior direction, and 0.7 mm in the superior‐inferior direction. With submillimeter agreement in each direction, it was determined that comparison between measurement and dose calculation could be accurately performed. The position of the V‐shaped organ at risk relative to the target was also confirmed to be 2 mm superior to the superior edge of the target.

### B. Treatment planning criteria

Development of the treatment planning criteria that institutions must meet in a quality assurance audit needed to provide clinically realistic challenges while being reasonably achievable given the phantom design. To address the latter point, the dose constraints on the OAR were relaxed from <1% of the OAR volume receiving 8 Gy to <10% of the OAR volume receiving 8 Gy. It was found that the close proximity of the OAR to the target (2 mm spacing) made it unreasonably difficult to deliver an adequate dose to the target while sparing the mock OAR of excess dose under the original definition. While this may not match the clinical criteria exactly, it was felt that, for the purpose of a quality assurance audit, a participating institution's ability to meet predefined dose constraints would still be adequately tested.

### C. Phantom dosimetry reproducibility

The phantom was irradiated three times with each radiosurgery system, to test for reproducibility. The calculated coefficient of variation, shown in Table 2, for the averaged TLDs were ±2.1%,±0.9%, and ±0.1%, for Gamma Knife, CyberKnife, and linac‐based deliveries, respectively. These numbers were low enough to imply acceptable reproducibility in an auditing and credentialing tool.

In terms of dose agreement, performance of Gamma Knife and CyberKnife irradiations showed average measured to TPS‐calculated dose ratios of 0.96 and 1.02, respectively. The measured to TPS‐calculated dose ratio was 0.94 for the linac‐based delivery and was outside the proposed ± 5% credentialing criterion. Closer inspection of the individual TLDs showed that the right anterior‐inferior TLD was located in a region of steep dose falloff for the linac plan in which even a small shift of 1 mm or 2 mm will result in unacceptable underirradiating of the TLD. The average measured to reported value of 0.91 in this specific TLD reflects this. While this error would result in a failure for the audited institution, the small coefficient of variation in the linac deliveries still supports the high degree of reproducibility in phantom performance that this study aimed to show.

Gamma analysis^(25)^ of the coronal and sagittal film planes using a global criterion of ±5%/3mm yielded coefficients of variation displayed in Table 3 of ±0.1% and ±1.0% in the coronal planes for Gamma Knife and CyberKnife deliveries, respectively, and ±0.7% and ±0.2% in the sagittal plane for Gamma Knife and CyberKnife deliveries, respectively. As with the absolute dose, this low coefficient of variation indicates a high degree of reproducibility, which was desired for this phantom.

**Table 2:** Reproducibility results for TLD measurements in three irradiations of the phantom using Gamma Knife, CyberKnife, and linac delivery systems

	*Averaged TLD Results*		
*Radiosurgery System*	*Meas./Reported*	σ	%σ
Gamma Knife	0.96	0.02	2.1%
CyberKnife	1.02	0.01	0.9%
Linac	0.94	0.001	0.1%

In terms of absolute gamma rates, the passing rates for Gamma Knife and CyberKnife deliveries were high, averaging 99.7% and 99.2%, respectively.

**Figure 4:**
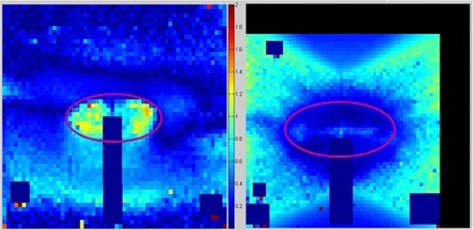
Gamma maps taken from coronal films of CyberKnife irradiation (left) and Gamma Knife irradiation (right) are shown. The overlaid purple outline represents the target location.

A visual inspection of the gamma maps identified a general trend of the areas where pixels were nearest the failing criteria for Gamma Knife and CyberKnife deliveries, shown in Fig. 4. The Gamma Knife showed the peripheral space surrounding the target to be closer to exceeding the ±5%/3mm criteria, while CyberKnife appeared to be more challenged at the very edges of the target. While these regions showed systematic trends in being nearer to the maximum allowable difference, the overwhelming majority of the pixels were still within the passing criteria, as shown in Table 4.

**Figure 5:**
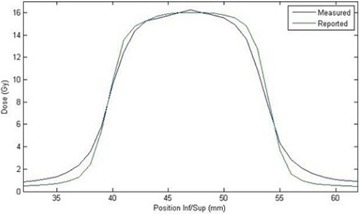
Dose profiles of film measurement (blue) and TPS reported (green) doses from linac‐based delivery along the superior–inferior direction of the coronal film plane. Position coordinates correspond to those established by the TPS.

**Figure 6:**
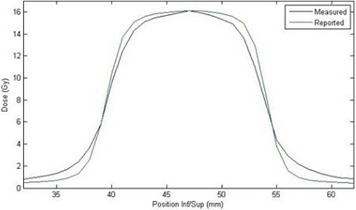
Dose profiles of film measurement (blue) and TPS reported (green) doses from linac‐based delivery along the superior–inferior direction of the sagittal film plane.

Because the institution's TPS was unable to export the plan in DICOM/RT format, distance‐to‐agreement criteria were used for evaluation of the linac‐based plan. The dose planes of interest were exported as ASCII files and compared to film manually in MATLAB, as shown in Figs. 5 and 6. DTA for the superior and inferior edges of the coronal and sagittal films, presented in Table 3, were 1.2 mm, 1.3 mm, 1.2 mm, and 1.2 mm, respectively. DTAs were measured along these profile edges due to the superior placement of the organ at risk with respect to the target in the imaging insert.

**Table 3:** Reproducibility results for gamma analysis in three irradiations of the phantom using Gamma Knife and CyberKnife delivery systems

	*Gamma Analysis* (±5%/3mm)					
	*Coronal Film*			*Sagittal Film*		
*Radiosurgery System*	*Passing Pixels*	σ	%σ	*Passing Pixels*	σ	%*G*
Gamma Knife	99.9%	0.1	0.1%	99.4%	0.7	0.7%
CyberKnife	98.8%	1.0	1.0%	99.5%	0.2	0.2%

**Table 4:** Reproducibility results for distance‐to‐agreement measurements in three irradiations of the phantom using a linac‐based delivery system

	*Inferior Edge*		*Superior Edge*	
*Film Plane*	*DTA (mm)*	*Std. Dev. (mm)*	*DTA (mm)*	*Std. Dev. (mm)*
Sagittal	1.2	1.1	1.3	1.0
Coronal	1.2	1.1	1.2	1.0

## IV. CONCLUSIONS

Modifications made to the imaging insert of a previously developed head phantom have provided a realistic clinical planning and delivery challenge to audited institutions. The inclusion of a target and critical structure that mimic real human anatomy provide for a more clinically relevant test when assessing institutions ability to deliver consistent and accurate radiation doses. The improved design and modified dose specifications have provided a thorough means of evaluating stereotactic radiosurgery from start to finish.

Reproducibility tests on the phantom using three means of treatment delivery, Gamma Knife radiosurgery system, CyberKnife robotic radiosurgery system, and standard linear accelerator delivery, showed the phantom performed in a consistent manner. Results indicate that the phantom provides a high degree of reproducibility under carefully controlled conditions. This demonstrates the usefulness of the phantom as a quality assurance tool in the evaluation of treatment accuracy for improving patient safety and the credentialing of institutions participating in clinical trials utilizing stereotactic radiosurgery.

## ACKNOWLEDGMENTS

This study was funded by grants from the National Cancer Institute (CA01093), Radiation Therapy Oncology Group (CA21661), and Advanced Technology Consortium (CA081647).
